# Temporal trends in the incidence and mortality of kidney cancer across BRICS from 1990 to 2021: an age-period-cohort analysis

**DOI:** 10.3389/fpubh.2025.1614193

**Published:** 2025-10-21

**Authors:** Yikai Wang, Zhengkun Wang, Weibing Shuang

**Affiliations:** ^1^The First Clinical Medical College, Shanxi Medical University, Taiyuan, China; ^2^Department of Urology, The First Hospital of Shanxi Medical University, Taiyuan, China

**Keywords:** kidney cancer, BRICS member countries, age-period-cohort mode, Global Burden of Disease, relative risk

## Abstract

**Introduction:**

This study aimed to systematically analyze the spatiotemporal heterogeneity and drivers of the kidney cancer burden across BRICS member countries (Brazil, Russia, India, China, South Africa, Egypt, Ethiopia, Indonesia, Iran, Saudi Arabia, and the United Arab Emirates) from 1990 to 2021. Given the significant global rise in kidney cancer incidence, elucidating its epidemiological characteristics and influencing factors in countries at different stages of economic development is crucial for formulating targeted prevention and control strategies.

**Methods:**

This study innovatively integrated Joinpoint regression and Age-Period-Cohort (APC) modeling using data from the Global Burden of Disease (GBD) 2021 study to quantify trends in kidney cancer incidence and mortality in BRICS nations. The APC model was employed to disentangle the independent effects of age, period, and birth cohort on the disease burden. These findings were subsequently interpreted in the context of national socioeconomic conditions and health policies to identify key drivers.

**Results:**

Globally, kidney cancer incidence increased by 142.74% from 1990 to 2021. Saudi Arabia experienced the most dramatic increase (877.78%), while Russia reported the highest Age-Standardized Incidence Rate (ASIR) in 2021 (10.10 per 100,000). Global mortality rates increased by 108.22%, led by the United Arab Emirates (700% growth), with Russia exhibiting the highest Age-Standardized Mortality Rate (ASMR) in 2021 (4.07 per 100,000). Furthermore, APC analysis identified critical drivers: the age effect peaked in the older populations (e.g., Russia’s mortality rate reached 42.8 per 100,000 at age 92.5); the period effect showed a surge in risk after 2000 in most nations (Saudi Arabia’s period Rate Ratio [RR]: 1.52); the cohort effect indicated a 6.60-fold elevated risk for China’s 2002 birth cohort compared to the 1952 baseline, contrasting with declining risks in younger Russian cohorts (RR: 0.66). Regional disparities highlighted interactions between economic transitions and health inequities. Specifically, Saudi Arabia’s burden was associated with metabolic disorders, Russia’s decline aligned with tobacco control initiatives, and India’s rural underdiagnosis reflected critical healthcare gaps.

**Discussion:**

The kidney cancer burden in BRICS countries demonstrates significant spatiotemporal heterogeneity, driven by mechanisms related to population aging, the prevalence of metabolic risk factors, and disparities in healthcare accessibility. The findings underscore the necessity of strengthening environmental governance, implementing metabolic health interventions, and optimizing healthcare resource allocation to advance health equity and achieve Sustainable Development Goals. These insights provide a scientific basis for developing cross-regional strategies for cancer control.

## 1 Introduction

As is well documented, kidney cancer has emerged as a critical global health challenge, with its incidence and mortality rates demonstrating a marked upward trajectory over the past three decades, particularly in rapidly developing economies undergoing profound demographic and socioeconomic transitions (1). The BRICS member countries (Brazil, Russia, India, China, South Africa, Egypt, Ethiopia, Indonesia, Iran, Saudi Arabia and the United Arab Emirates), representing over 40% of the global population, are experiencing concurrent industrialization, urbanization, aging, and lifestyle shifts that profoundly reshape cancer epidemiology (2, 3). Existing studies under the Global Burden of Disease (GBD) framework have documented worldwide trends in kidney cancer, yet systematic analyses focusing on BRICS member countries, a group marked by economic growth and health inequities, remain scarce. Prior research has predominantly isolated individual risk factors (e.g., obesity, smoking) or regional data without accounting for the synergistic interactions of age, period, and cohort effects in shaping long-term temporal trends (4, 5). For instance, Saudi Arabia experienced an 877.78% surge in kidney cancer incidence from 1990 to 2021, while Russia’s persistently high mortality rates reflect systemic disparities in healthcare access. In China, rising risks among younger cohorts may stem from transgenerational impacts of industrial pollution and metabolic disorders (6, 7). However, the mechanisms underlying these patterns, such as cumulative exposure to environmental toxins during economic transitions, differential policy impacts on early diagnosis, and intergenerational shifts in lifestyle behaviors, have not been comprehensively modeled.

This study innovatively integrated Joinpoint regression and age-period-cohort (APC) analysis using GBD 2021 data to systematically quantify the spatiotemporal heterogeneity of kidney cancer burden and its drivers across BRICS member countries. Methodologically, conventional epidemiological approaches generally overlook collinearity among age, period, and cohort variables, leading to biased attribution of risk factors. For example, underdiagnosis in rural India due to fragmented screening infrastructure likely underestimates true incidence rates, while Russia’s tobacco control policies reduced mortality among younger populations yet failed to alleviate the disease burden in aging cohorts (8–11). Furthermore, intra-BRICS disparities, such as divergent metabolic disease profiles between China and Saudi Arabia, as well as urban–rural healthcare inequalities in South Africa, highlight the need for a study design that balances global trends with regional specificity (12–14). By dissecting age-dependent risk escalation, period-specific policy efficacy, and cohort-level cumulative exposures, this research not only addresses evidence gaps in cancer control for emerging economies but also informs cross-regional strategies targeting environmental governance, metabolic health interventions, and healthcare resource optimization. In the context of escalating non-communicable disease burdens globally, these findings hold urgent implications for advancing health equity and sustainable development goals (15).

## 2 Materials and methods

### 2.1 Data sources

The data employed in this study were sourced from the Global Health Data Exchange GBD Results Tool.^1^ The GBD 2021 study provides the most recent and comprehensive descriptive epidemiological data for 288 causes of death, 371 diseases and injuries, and 88 risk factors across 204 countries and territories from 1990 to 2021. We extracted data on the incidence, mortality, and the corresponding age-standardized incidence rate (ASIR) and age-standardized mortality rate (ASMR) for kidney cancer in BRICS member countries and globally from the GBD database. All estimates were reported in 95% uncertainty intervals (UIs), which were sampled repeatedly 1,000 times, with upper and lower bounds based on the 2.5th and 97.5th percentiles of the uncertainty distribution. The average annual percentage change (AAPC) and its 95% confidence interval (95% CI) were calculated using Joinpoint software (National Cancer Institute, Rockville, MD, USA) to assess the trend in disease burden. The data utilized in this study were anonymized and publicly available.

### 2.2 Statistical analysis

#### 2.2.1 Joinpoint regression analysis

The Joinpoint regression model is a statistical method that analyses the trend of the incidence of disease or mortality over time. The model initially fits the original dataset using the minimum number of joinpoints, specifically zero joinpoints, which corresponds to a straight line. Subsequently, it evaluates whether the inclusion of additional joinpoints is statistically justified and should be incorporated into the model. The maximum permissible number of joinpoints is contingent upon the input data. In this study, the number of observations is at least 29, which by default allows for a maximum of four joinpoints, thereby partitioning the data into five distinct trend segments and enhancing the credibility of the results. This investigation employed the Joinpoint regression analysis model to conduct a trend analysis on the incidence and mortality data of kidney cancer in BRICS member countries. The analysis utilized metrics such as the annual percent change (APC) and the AAPC to evaluate trends in the incidence and mortality of kidney cancer within these nations. An APC value exceeding zero signifies an upward trend in kidney incidence or mortality, whereas a value below zero indicates a downward trend. The AAPC value represents the geometrically weighted average of the APC values (16, 17). The statistical significance of each trend segment’s annual percent change values and the overall AAPC value was validated using the Monte-Carlo permutation method. All the above procedures were implemented using the Joinpoint regression program V.5.3.0.0 provided by the National Cancer Institute (NCI) of the USA. The current analysis was performed on the general population. Future research incorporating stratification by sex would be beneficial to investigate potential heterogeneity in risk patterns.

#### 2.2.2 Age-period-cohort analysis

Collinearity is a common issue in the application of the APC model, as it involves a relationship between cohort equals period minus age. In this study, we employed the intrinsic estimator (IE) algorithm, whose parameters have been proven to be estimable, unbiased, efficient and asymptotically normal (18, 19). In this study, the APC model was employed to analyze the data, with age, period, and cohort serving as independent variables. The model conceptualizes the incidence of the observed event or phenomenon within the population as the dependent variable, assuming an underlying probability distribution. By employing APC models, this research extends beyond traditional epidemiological analyses to elucidate the impact of various factors on disease trends. Within the APC framework, the age effect denotes the differential risk of outcomes across distinct age groups, the period effect captures temporal changes in outcomes that simultaneously affect all age groups, and the cohort effect reflects variations in outcomes among individuals born in the same year.

To manage the number of parameters in the APC model while achieving a smooth curve for time effects, this study categorized age-specific incidence rates into five-year intervals (15–19, 20–24, 25–29, …, 90–94 years). The APC model necessitates that both age and period be segmented into equal intervals; therefore, period data were similarly divided into five-year groups (1992–1996, 1997–2001, …, 2017–2021). The incidence and corresponding population data were consolidated into a cohesive framework, with all data averaged over five-year spans. This study primarily focuses on several estimable functions: the net drift, which represents the overall annual percentage change in incidence rates over time; local drifts, which detail the annual percentage changes by period and cohort for each age group; the longitudinal age curve, which depicts the fitted age-specific rates over time within the reference cohort, accounting for period deviations; and the period (or cohort) rate ratio (RR), which indicates the ratio of age-specific rates in each period (or cohort) relative to the reference period (or cohort). An RR value >1 implies an increased rate of kidney cancer incidence, while an RR value <1 suggests a decreased rate. The significance of the estimated parameters and functions was assessed using the Wald chi-squared test, with all statistical tests conducted as two-tailed.

#### 2.2.3 Ethical information

Data were all analyzed anonymously, so ethical approval was not needed. All methods in this paper were performed following the relevant guidelines and regulations.

## 3 Results

### 3.1 Global and BRICS member countries’ trends in kidney cancer incidence and mortality

**Figure 1:**
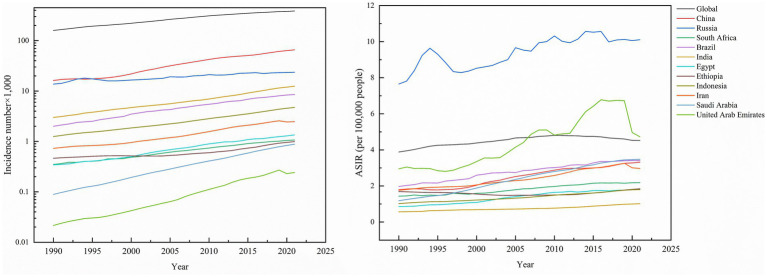
Incidence number of kidney cancer, and ASIR of kidney cancer across global and BRICS member countries between 1990 and 2021.

Globally, kidney cancer incidence surged from 159,770 cases (95% UI: 154,830–163,930) in 1990 to 387,830 cases (95% UI: 365,360–406,640) in 2021, marking a 142.74% cumulative increase. Among the 11 BRICS member countries analyzed, Saudi Arabia experienced the most dramatic rise (877.78%), followed by Brazil (327.00%), India (313.29%), and Indonesia (278.40%), while Russia exhibited the smallest growth (71.73%). The global age-standardized incidence rate (ASIR) rose from 3.89 (95% UI: 3.76–3.99) to 4.52 (95% UI: 4.26–4.75) per 100,000 population, with Saudi Arabia demonstrating both the steepest absolute ASIR surge (192.44%) and the highest average annual percentage change (AAPC) of 3.50% (95% CI, 3.35–3.64). By 2021, Russia reported the highest national ASIR (10.10 per 100,000; 95% UI: 9.29–10.93), while India recorded the lowest (1.02 per 100,000, 95% UI: 0.91–1.13). Notably, all countries showed positive AAPC trends, ranging from Ethiopia’s modest 0.30% (95% CI, 0.20–0.40) to Egypt’s significant 2.46% (95% CI, 2.21–2.71), reflecting universal upward trajectories in kidney cancer burden over the three decades (Table 1; Figure 1).

**Table 1:** Characteristics of kidney cancer incidence and death in BRICS member countries between 1990 and 2021.

Characteristic	Global		China		South Africa		Russia		India		Brazil	
	1990	2021	1990	2021	1990	2021	1990	2021	1990	2021	1990	2021
Population												
Number, *n* × 1,000,000^a^	5333.62 (5231.04–5444.65)	7891.35 (7666.73–8131.23)	1176.46 (1096.74–1263.98)	1422.75 (1318.76–1530.46)	37.01 (33.16–40.94)	56.85 (49.73–64.28)	150.99 (138.99–162.54)	144.85 (124.99–163.58)	853.07 (788.85–914.90)	1414.50 (1240.24–1601.51)	148.51 (137.93–158.98)	220.36 (188.14–250.95)
Percentage of global, %	100.00	100.00	22.06	18.03	0.69	0.72	2.83	1.84	16.00	17.92	2.78	2.79
Incidence												
Number, *n* × 1,000^a^	159.77 (154.83–163.93)	387.83 (365.36–406.64)	16.23 (14.23–18.29)	65.80 (53.69–79.74)	0.76 (0.69–0.84)	2.22 (1.74–2.68)	13.76 (13.42–14.12)	23.63 (21.68–25.60)	3.01 (2.69–3.36)	12.44 (11.11–13.85)	2.00 (1.92–2.09)	8.54 (8.01–8.99)
Percentage of global, %	100.00	100.00	10.16	16.97	0.48	0.57	8.61	6.09	1.89	3.21	1.25	2.20
Percent change of incidence 1990–2021, %	142.74		305.42		192.11		71.73		313.29		327.00	
Age-standardized incidence rates												
Rate per 100,000^a^	3.89 (3.76–3.99)	4.52 (4.26–4.75)	1.79 (1.58–2.01)	3.32 (2.72–3.98)	1.43 (1.22–1.64)	2.19 (1.98–2.37)	7.65 (7.46–7.85)	10.10 (9.29–10.93)	0.56 (0.51–0.63)	1.02 (0.91–1.13)	1.97 (1.88–2.05)	3.41 (3.20–3.59)
Percent change of rate 1990–2021, %	16.20		85.48		53.15		32.03		82.14		73.10	
AAPC, %^b^	0.49 (0.42–0.56)		2.03 (1.61–2.45)		1.33 (1.11–1.56)		1.00 (0.49–1.50)		1.95 (1.77–2.13)		1.85 (1.49–2.22)	
Death												
Number, *n* × 1,000^a^	77.42 (74.81–79.69)	161.20 (150.32–169.35)	9.05 (7.94–10.17)	24.87 (20.36–29.83)	0.54 (0.49–0.59)	1.41 (1.12–1.69)	6.66 (6.49–6.84)	9.83 (9.01–10.65)	2.28 (2.05–2.54)	8.05 (7.15–8.96)	1.34 (1.28–1.40)	5.01 (4.66–5.29)
Percentage of global, %	100.00	100.00	11.69	15.43	0.70	0.88	8.60	6.10	2.94	5.00	1.70	3.11
Percent change of death 1990–2021, %	108.22		174.81		161.11		47.60		253.10		273.88	
Age-standardized deaths rates												
Rate per 100,000^a^	1.99 (1.91–2.06)	1.91 (1.78–2.01)	1.14 (1.00–1.28)	1.25 (1.03–1.48)	1.06 (0.87–1.23)	1.44 (1.31–1.55)	3.68 (3.58–3.78)	4.07 (3.73–4.42)	0.47 (0.42–0.52)	0.69 (0.61–0.77)	1.43 (1.36–1.50)	2.02 (1.87–2.13)
Percent change of rate 1990–2021, %	−4.02		9.65		35.85		10.60		46.81		41.26	
AAPC, %^a^	−0.13 (−0.23 to −0.04)		0.22 (−0.11–0.55)		0.95 (0.54–1.37)		0.41 (−0.18–1.00)		1.28 (1.06–1.51)		1.18 (0.82–1.54)	

Characteristic	Egypt		Ethiopia		Indonesia		Iran		Saudi Arabia		United Arab Emirates	
	1990	2021	1990	2021	1990	2021	1990	2021	1990	2021	1990	2021
Population												
Number, *n* × 1,000,000^a^	55.33 (50.23–60.51)	105.63 (95.74–115.67)	50.57 (46.30–55.70)	108.94 (91.83–125.47)	184.98 (172.49–197.57)	278.91 (256.92–300.11)	57.10 (51.97–61.98)	85.36 (76.86–93.93)	15.86 (14.48–17.16)	37.70 (32.63–43.02)	1.87 (1.69–2.05)	9.63 (7.90–11.22)
Percentage of global, %	1.04	1.34	0.95	1.38	3.47	3.53	1.07	1.08	0.30	0.48	0.04	0.12
Incidence												
Number, *n* × 1,000^a^	0.34 (0.30–0.41)	1.35 (1.08–1.68)	0.47 (0.29–0.72)	0.99 (0.69–1.32)	1.25 (1.00–1.48)	4.73 (3.83–5.82)	0.73 (0.60–0.88)	2.48 (2.24–2.74)	0.09 (0.07–0.12)	0.88 (0.67–1.11)	0.02 (0.02–0.03)	0.24 (0.19–0.31)
Percentage of global, %	0.21	0.35	0.29	0.26	0.78	1.20	0.46	0.64	0.06	0.23	0.01	0.06
Percent change of incidence 1990–2021, %	297.06		110.64		278.40		239.73		877.78		110.00	
Age-standardized incidence rates												
Rate per 100,000^a^	0.86 (0.77–0.97)	1.83 (1.50–2.23)	1.69 (1.31–2.21)	1.84 (1.22–2.49)	1.03 (0.84–1.19)	1.80 (1.46–2.21)	1.76 (1.56–2.04)	2.97 (2.69–3.28)	1.19 (0.89–1.54)	3.48 (2.76–4.26)	2.95 (2.10–4.18)	4.73 (3.77–5.79)
Percent change of rate 1990–2021, %	112.79		8.88		74.76		68.75		192.44		60.34	
AAPC, % ^a^	2.46 (2.21–2.71)		0.30 (0.20–0.40)		1.84 (1.77–1.90)		1.67 (1.46–1.88)		3.50 (3.35–3.64)		1.31 (0.25–2.37)	
Deaths												
Number, *n* × 1,000^a^	0.16 (0.15–0.18)	0.54 (0.44–0.66)	0.37 (0.25–0.56)	0.67 (0.45–0.91)	0.69 (0.56–0.81)	2.37 (1.90–2.92)	0.28 (0.26–0.32)	0.86 (0.78–0.94)	0.05 (0.04–0.06)	0.28 (0.21–0.36)	0.01 (0.01–0.01)	0.08 (0.06–0.10)
Percentage of global, %	0.10	0.34	0.48	0.42	0.89	1.47	0.36	0.53	0.07	0.17	0.01	0.05
Percent change of deaths 1990–2021, %	237.50		81.08		243.48		207.14		460.00		700.00	
Age-standardized deaths rates												
Rate per 100,000^a^	0.52 (0.47–0.59)	0.92 (0.76–1.11)	1.52 (1.19–1.97)	1.43 (0.93–1.96)	0.64 (0.52–0.74)	0.99 (0.79–1.21)	0.93 (0.83–1.04)	1.16 (1.04–1.27)	0.77 (0.56–1.01)	1.50 (1.20–1.85)	1.85 (1.33–2.50)	2.54 (2.01–3.08)
Percent change of rate 1990–2021, %	76.92		−5.92		54.69		24.73		94.81		37.3	
AAPC, %^a^	1.84 (1.51–2.18)		−0.18 (−0.26 to −0.10)		1.42 (1.34–1.51)		0.67 (0.50–0.83)		2.18 (2.06–2.30)		0.81 (−0.60–2.24)	

a The values in the parenthesis were 95% uncertain intervals.

b The values in the parenthesis were 95% confidence intervals.

**Figure 2:**
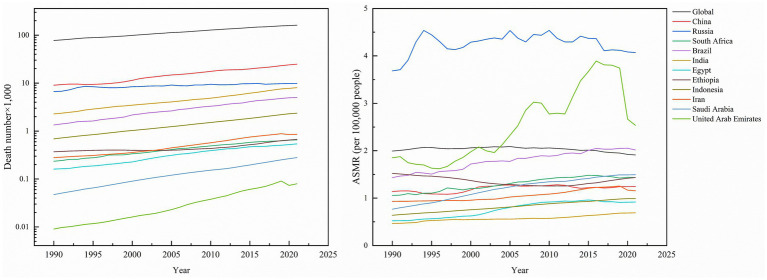
Death number of kidney cancer, and ASMR of kidney cancer across global and BRICS member countries between 1990 and 2021.

Global kidney cancer-related deaths rose from 77,420 (95% UI: 74,810–79,690) to 161,200 (95% UI: 150,320–169,350), a 108.22% increase. As anticipated, mortality growth varied substantially across regions, with the United Arab Emirates (UAE) leading at 700.00%, followed by Saudi Arabia (460.00%), Brazil (273.88%), and Indonesia (243.48%), while Russia experienced the smallest rise (47.60%). In contrast, the global age-standardized mortality rate (ASMR) declined by −4.02% to 1.91 per 100,000 population (95% UI: 1.78–2.01). Nationally, Ethiopia was the sole country with reduced ASMR (−5.92%), whereas Saudi Arabia saw the largest increase (94.81%). Russia maintained the highest ASMR in 2021 (4.07; 95% UI: 3.73–4.42), contrasting with India’s lowest rate (0.69; 0.61–0.77). Mortality AAPC trends were predominantly positive, peaking in Saudi Arabia (2.18%; 95% CI: 2.06–2.30) and Egypt (1.84%; 95% CI: 1.51–2.18), while the UAE was the only nation with a negative AAPC (−0.18%; 95% CI: −0.26 to −0.10), highlighting critical disparities in healthcare outcomes across regions (Table 1; Figure 2).

### 3.2 Net drift and local drift in different age groups

**Figure 3:**
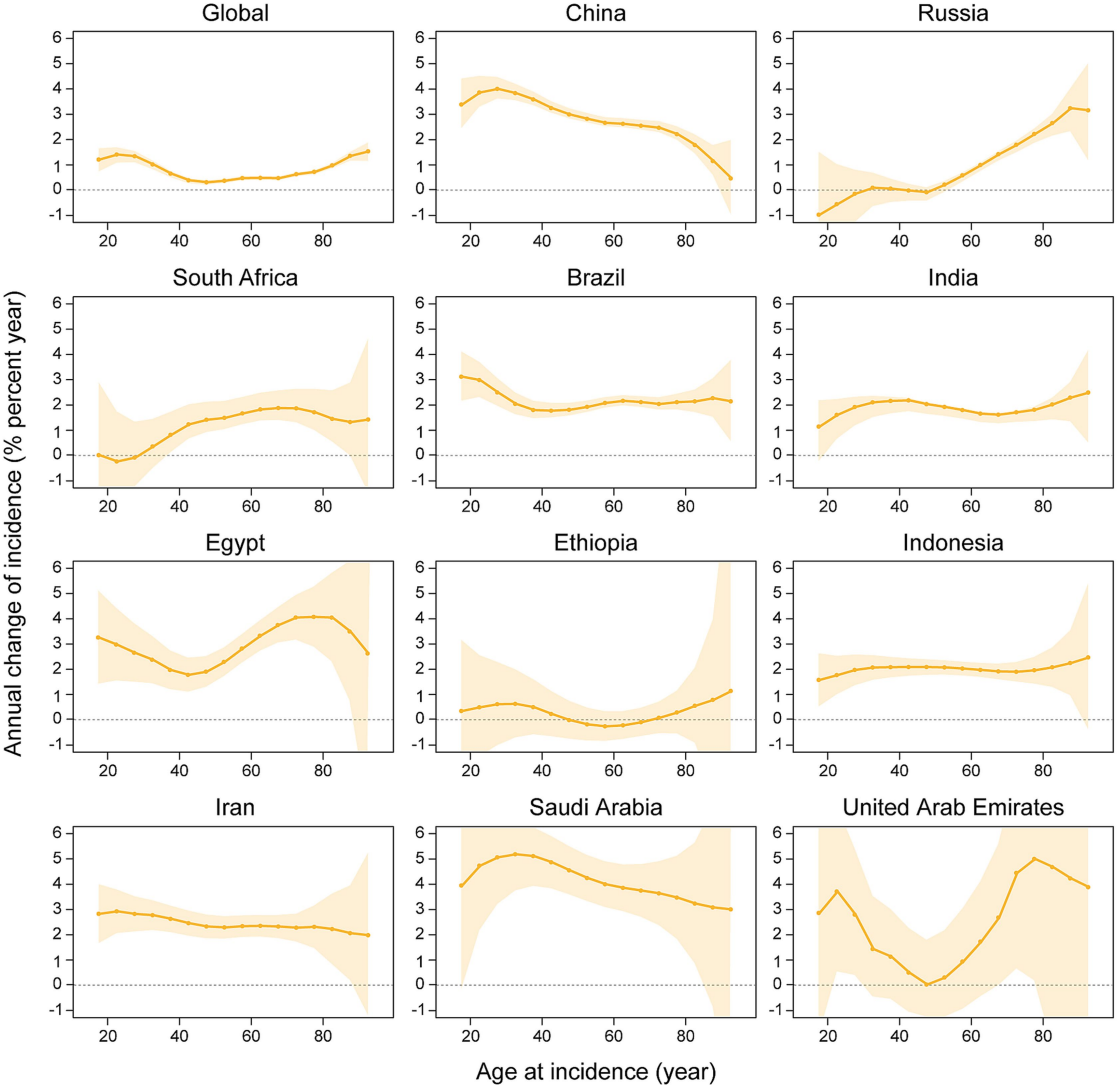
The local drifts and net drifts of kidney cancer incidence rate in global and BRICS member countries, 1992–2021 (The legend located in the upper right corner clearly identifies all the countries).

Net drift refers to the overall annual percentage change in incidence rates across the entire study period, whereas localized drift denotes the annual percentage change in incidence rates at each age, relative to the net drift. Figure 3 illustrates the annual percentage change in the incidence rate of kidney cancer across various age groups. The net drift analysis demonstrated a significant increase in kidney cancer incidence across all 11 studied nations and globally, with a worldwide net drift of 0.5805%/year (95% CI: 0.5285–0.6324). Among individual countries, Saudi Arabia exhibited the highest net drift (4.0569%/year; 95% CI: 3.2241–4.8964), followed by China (3.2232%/year; 95% CI: 3.0826–3.3641), Iran (2.593%/year; 95% CI: 2.2209–2.9664), Egypt (2.9513%/year; 95% CI: 2.4068–3.4987), and Brazil (1.9756%/year; 95% CI: 1.7849–2.1666). On the other hand, India, Indonesia, South Africa, and the United Arab Emirates showed moderate increases (1.9291%/year to 2.5298%/year), while Russia and Ethiopia displayed lower drifts (0.5734%/year and 0.41%/year, respectively; Ethiopia’s CI includes zero). Age-specific local drift analysis revealed divergent patterns globally: younger groups (17.5 years: 1.0826%/year) and the older population (92.5 years: 1.5334%/year) showed rising incidence rates, whereas middle-aged groups (47.5 years: 0.0427%/year) remain stable. Of note, China exhibited the highest local drift in young adults (27.5 years: 4.7279%/year), whereas a decline was noted among the oldest cohort (92.5 years: −0.1124%/year). In Russia, a decline in net drift was observed in younger individuals (17.5 years: −1.5081%/year), whereas an increase was identified in the older population (92.5 years: 3.85%/year). More importantly, individuals aged ≥90 years exhibited increasing incidence across all nations, with the exception of China.

**Figure 4:**
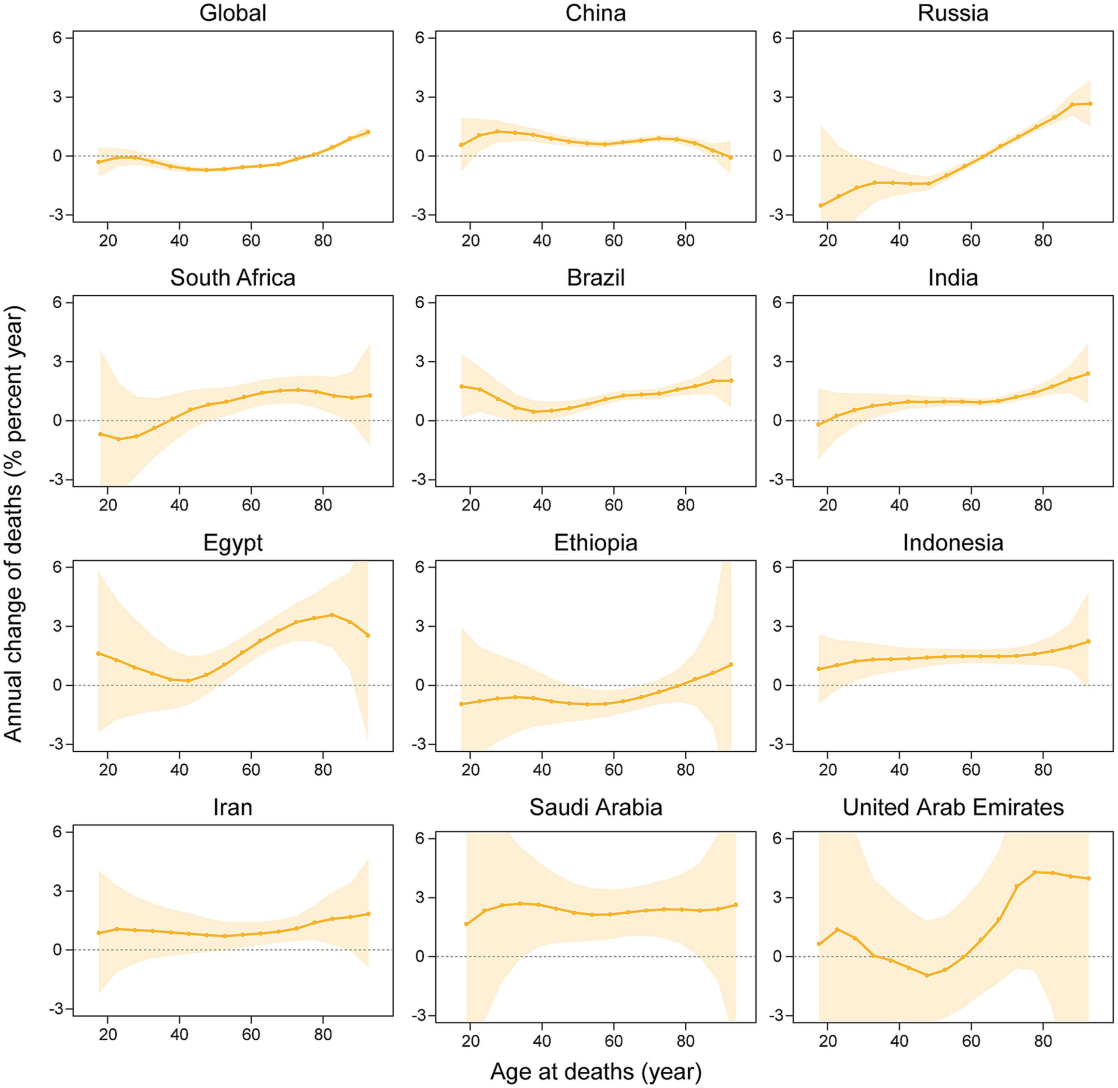
The local drifts and net drifts of kidney cancer mortality rate in global and BRICS member countries, 1992–2021 (The legend located in the upper right corner clearly identifies all the countries).

Figure 4 delineates the annual percentage change in the mortality rate of kidney cancer across age groups, with significant heterogeneity in mortality drift across BRICS member countries and globally. Saudi Arabia (net drift +2.18%/year; 95% CI: 0.92–3.46), Egypt (+1.72%/year; 0.93–2.52), and Indonesia (+1.41%/year; 1.07–1.76) exhibited the most pronounced increase, with consistently elevated local drifts (e.g., +2.06%/year at age 62.5 in Saudi Arabia and +3.78%/year at age 82.5 in Egypt). Brazil (+1.07%/year; 0.80–1.34), China (+0.95%/year; 0.80–1.10), India (+0.94%/year; 0.70–1.18), and Iran (+1.17%/year; 0.56–1.78) exhibited steady growth, with India and Indonesia demonstrating age-amplified local drifts (e.g., +2.12%/year at age 92.5 in India). Russia (−0.74%/year; −1.06–-0.43) and global average (−0.38%/year; −0.45–-0.32) significantly declined, marked by sharp reductions in younger Russian cohorts (−3.26%/year at age 17.5) and rising trends in older groups (+3.27%/year at age 92.5). South Africa (+0.76%/year; 0.13–1.39) and the UAE (+1.39%/year; −1.30–4.14) displayed fluctuating patterns, with extreme local drift variability in the UAE (−1.07%/year at age 52.5 vs. + 6.41%/year at age 92.5). Ethiopia (−0.42%/year; −1.15–0.31) displayed a non-significant decline, with predominantly negative local drifts across age groups (e.g., −0.85%/year at age 52.5).

### 3.3 Age-period-cohort effects on kidney cancer incidence rate and mortality rate

**Figure 5:**
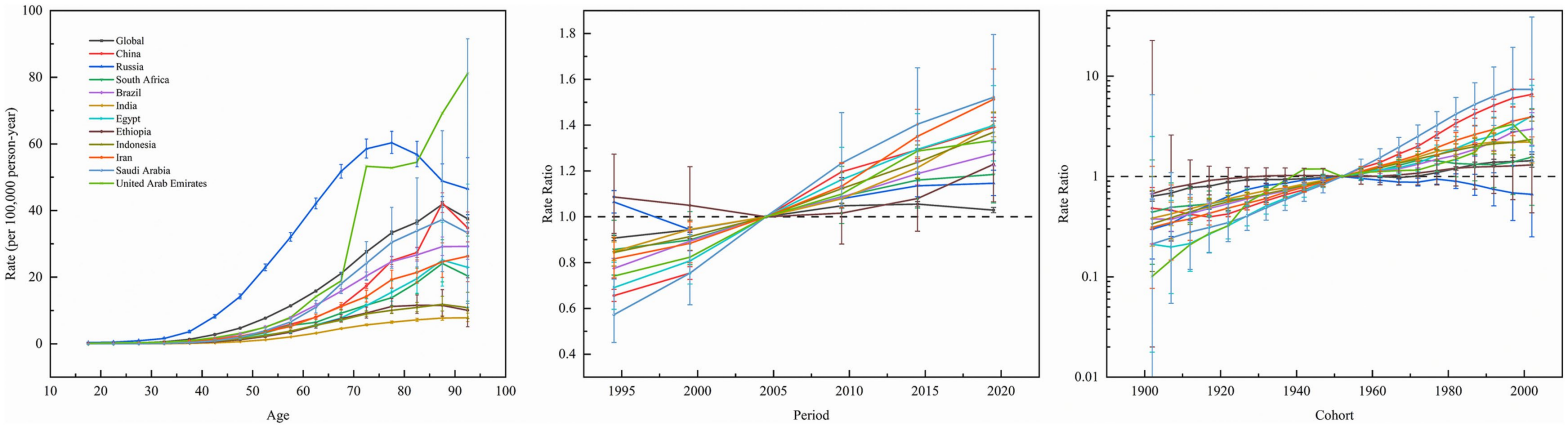
Age, period and cohort effects on kidney cancer incidence in global and BRICS member countries (The data from the United Arab Emirates has a very broad range of uncertainty, impacting its representativeness, so it was not included. The legend located in the upper left corner clearly identifies all the countries).

Figure 5 depicts the effect of age, period, and cohort effects on the incidence of kidney cancer. The APC analysis of kidney cancer incidence across 11 BRICS member countries and global aggregates uncovered marked heterogeneity in age-specific vulnerabilities, temporal trends, and generational risk patterns. Age-specific rates universally increased with advancing age, ranging from minimal levels in young adults (e.g., India: 0.0075/100,000 at age 17.5; Saudi Arabia: 0.0112/100,000 at age 17.5) and peaked among the older population (e.g., Russia: 60.4/100,000 at age 77.5; Global: 41.87/100,000 at age 87.5), with the United Arab Emirates exhibiting a unique trajectory of low youth incidence (age 17.5: 0.1931/100,000) and the highest older population burden globally (age 72.5: 53.29/100,000). Meanwhile, period effects demonstrated consistent post-2000 increases with variable magnitudes. For instance, Saudi Arabia exhibited the steepest rise (2019.5 vs. 2004.5 period ratio: 1.52, 95% CI: 1.29–1.80), followed by Egypt (1.40, CI, 1.24–1.57), China (1.39, CI, 1.35–1.43), Brazil (1.27, CI, 1.22–1.33), and Russia (1.15, CI: 1.09–1.20). Cohort effects highlighted divergent generational trajectories. For example, China’s 2002 birth cohort exhibited a 6.60-fold risk elevation (CI: 4.68–9.30) compared to the 1952 baseline cohort, paralleled by elevated risks in Egypt (2002 cohort RR: 4.03, CI: 2.01–8.09) and Iran (2002 cohort RR: 3.94, CI: 2.48–6.26). Conversely, Russia’s younger cohorts showed declining risk (2002 cohort RR: 0.66, CI: 0.25–1.76), while Ethiopia maintained minimal cohort variation.

**Figure 6:**
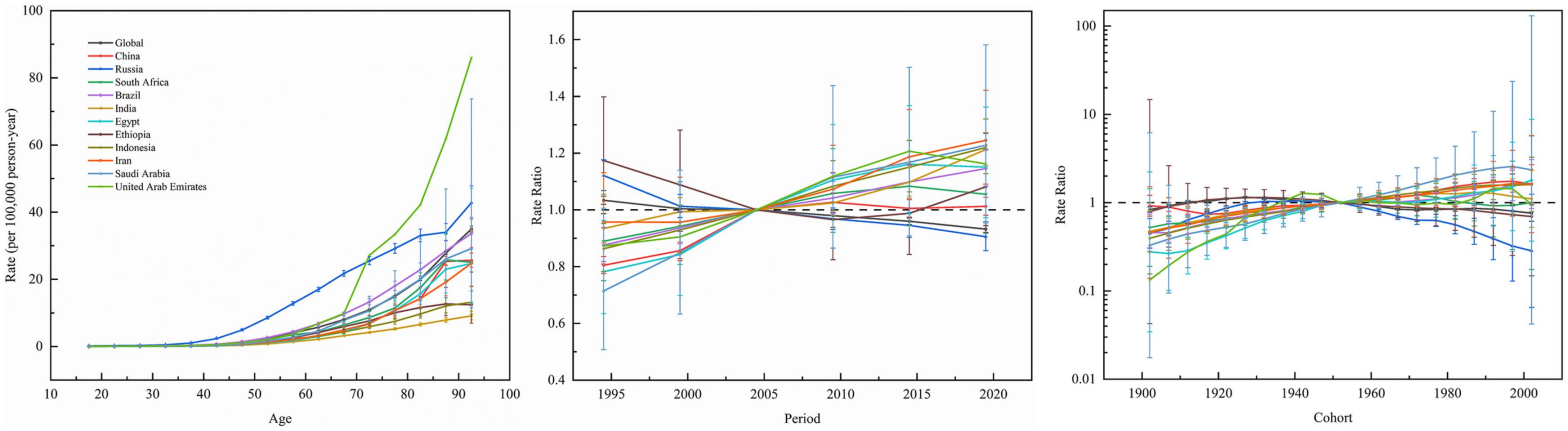
Age, period and cohort effects on kidney cancer mortality in global and BRICS member countries (The data from the United Arab Emirates has a very broad range of uncertainty, impacting its representativeness, so it was not included. The legend located in the upper left corner clearly identifies all the countries).

Figure 6 shows the effect of age, period, and cohort effects on the mortality of kidney cancer. Across BRICS member countries and globally, kidney cancer mortality exhibited a consistent age-dependent increase, rising from minimal levels in youth (e.g., 0.0059/100,000 at age 17.5 in India) to peak rates in the older population (e.g., 42.8/100,000 at age 92.5 in Russia). Period effects diverge markedly. Specifically, Brazil, China, Egypt, India, Indonesia, Iran, and Saudi Arabia showed upward trends, with risk ratios (RR) peaking between 2014.5–2019.5 (e.g., Iran: RR = 1.2453 in 2019.5; Saudi Arabia: RR = 1.2282 in 2019.5). In contrast, Russia and global data demonstrated declining period effects (Russia: RR = 0.9056 in 2019.5; Global: RR = 0.9322 in 2019.5), while Ethiopia and South Africa exhibited minimal fluctuations (RR range: 0.8897–1.1742). Cohort effects revealed generational shifts, that is, younger cohorts (post-1952) in China (1997 cohort: RR = 1.7514), Egypt (2002 cohort: RR = 1.7955), India, Indonesia, Iran, and Saudi Arabia (1997 cohort: RR = 2.5718) faced elevated risks, whereas declining risks were observed in Ethiopia (2002 cohort: RR = 0.6966), Russia (2002 cohort: RR = 0.2849), and globally (2002 cohort: RR = 0.7605).

## 4 Discussion

Previous epidemiological studies based on the GBD framework have predominantly concentrated on documenting global or regional trends in kidney cancer incidence and quantifying the contributions of various risk factors. However, comprehensive investigations into the long-term dynamic mechanisms, particularly the interactions of APC in emerging economies, remain lacking. For instance, while earlier analyses of GBD 2021 data highlighted the increasing burden of kidney cancer among aging populations (those aged 65 years and older), these studies did not employ APC models to disentangle intergenerational risk trajectories or the effects of cumulative exposure (4). Similarly, studies identifying metabolic syndrome as a significant driver of kidney cancer in China has frequently neglected to consider the critical interactions between industrial-era environmental pollutants (such as heavy metals and airborne particulates) and cohort-specific susceptibility patterns (5). Furthermore, although studies have quantified population-attributable risks for biomarkers such as high body mass index (BMI), these approaches typically overlook systematic assessments of disparities in healthcare accessibility and the impacts of policy across BRICS member countries (3). The present study integrated Joinpoint regression for temporal trend segmentation with APC modeling within the GBD framework to elucidate transgenerational factors, ranging from the legacy effects of industrial pollution to disparities in healthcare systems, that influence kidney cancer trajectories in BRICS member countries.

Differences in kidney cancer burden among BRICS member countries highlight the complex interplay between economic development, public health policies, and societal structures. Despite large populations and rising absolute case numbers, China and India showed relatively moderate growth in ASIR, which can be ascribed to improved primary healthcare and cancer screening programs, though long-term risks from industrialization-related pollution and lifestyle changes require surveillance (20–22). Russia, South Africa, and Brazil exhibited high ASIR and mortality rates, reflecting systemic healthcare inequities and insufficient control of risk factors such as smoking and obesity, compounded by delayed diagnoses in remote areas (23–28). At the same time, Middle Eastern nations such as Saudi Arabia and the UAE faced rapid ASIR increases, driven by urbanization-induced metabolic diseases and a healthcare focus on tertiary care over preventive measures (29–31). Egypt and Ethiopia’s higher mortality relative to incidence highlight inadequate medical infrastructure and prevalent late-stage diagnoses (32–34). Noteworthily, Indonesia and Iran’s rising burden was correlated with industrial pollution and occupational exposures (35–38). Policy efforts should prioritize cross-regional collaboration, integrating cancer control into national health strategies. This includes utilizing economic tools (e.g., tobacco/alcohol taxation), and rebalancing healthcare resources to bridge urban–rural gaps. Besides, public health initiatives must address regional priorities: lifestyle interventions in high-income countries, grassroots screening capacity in low-resource settings, and transnational environmental governance with enhanced data transparency.

The APC analysis of kidney cancer incidence uncovered key public health challenges and policy implications across BRICS member countries. The age effect’s inverted U-curve (e.g., Russia’s peak rate of 56.8/100,000 at 82.5 years) universally positions aging as a key driver, yet divergent peaks (Brazil 26.7/100,000 vs. Saudi Arabia 37.3/100,000) signal inequitable geriatric healthcare access (39–41). Period effects revealed critical divergence: steep post-2004 surges in India (2019 period RR 1.40), Indonesia (1.37), Iran (1.51), and China (39% rise) directly mirror uncontrolled risk behaviors during economic transitions, including increased processed food consumption, sedentary lifestyles, and delayed tobacco control, highlighting prevention systems lagging behind growth (27, 42, 43). Conversely, Russia’s declining period and cohort trends are temporally consistent with the implementation of its comprehensive tobacco control policies, including the 2008 indoor smoking ban and earlier tax reforms. Our findings suggest that this successful intervention may have contributed to altering the kidney cancer risk trajectory, particularly among younger cohorts. This offers a transferable lesson: timely policies have the potential to disrupt the ‘development-disease burden’ cycle (39). Cohort effects exposed intergenerational crises: higher risks in younger Saudi (1987 Cohort RR: 5.23), Chinese (4.22), and Egyptian (2.29) cohorts indicate lifelong impacts of childhood obesity and environmental toxins, with Saudi Arabia’s extreme post-1952 cohort risk (Cohort RR: 7.4), aligning with a high prevalence of adult obesity at 70%and soft drink intake exceeding 120 L/year, unmasking a “resource curse” paradox where economic prosperity promotes the development of metabolic disease. While our ecological analysis cannot prove causation, the temporal association between the nation’s rapid economic transformation, the emergence of these risk factors, and the steep rise in kidney cancer incidence is compelling. This underscores the urgent need for policy interventions targeting metabolic health, such as regulations on ultra-processed foods and sugar-sweetened beverages, whose effectiveness has been demonstrated in other settings (44–46). The distinctive intergenerational protection observed in Russia, exemplified by a Cohort Relative Risk of 0.85 in 1937, is indicative of historical influences, such as the food shortages experienced during the Soviet era that curtailed risky behaviors. However, the annual local drift rate among the older population still escalated to 2.84%, highlighting the inadequacy of a singular policy approach in addressing the multifaceted challenges present. Data gaps reflect systemic challenges. Ethiopia’s near-unity cohort effects (Cohort RR: 1.24) indicate underdiagnosis due to primary care deficits, while the UAE’s wide CIs (e.g., 72.5-year rate 53.3, CI 31.8–89.4) reflect that migrant labor dynamics destabilize surveillance, necessitating targeted public health investments (47, 48). Policy actions demand tiered responses. High-risk nations (China, India, Saudi Arabia) must elevate childhood obesity prevention to a national strategic priority, adopting measures such as Mexico’s “soda tax,” which resulted in a 12% reduction in obesity prevalence, and Brazil’s school meal programs, coupled with industrial pollutant regulation (49, 50). Russia’s tobacco control model should guide Iran and Indonesia, using fiscal pivots (e.g., diverting Indonesia’s palm oil subsidies to screening centers). Furthermore, Egypt and Ethiopia may benefit from mobile screening programs in rural areas (modeled on India’s National Mobile Health Units) and dietary education delivered via religious networks. The global older population burden (local drift >1.2%/year for ages 80+) requires the integration of geriatric kidney cancer management into universal health coverage systems, supported by GP-led “frailty index” monitoring. Fundamentally, the APC model exposes a development paradox: economic growth without proportional preventive health investment ultimately manifests as an elevated disease burden. BRICS member countries must establish integrated cancer burden accounts within their national GDP accounting systems to incorporate healthcare and break the cycle linking cancer, health expenditure, and economic output.

Analysis of APC models for kidney cancer mortality in BRICS member countries and globally (excluding drift effects) offered critical insights into the interplay of aging, temporal exposures, and generational risks. Age effects were most pronounced in older populations (e.g., Brazil’s mortality rate of 13.28/100,000 in ages ≥72.5), highlighting cumulative environmental toxins (e.g., industrial pollutants) and comorbidities (e.g., hypertension), necessitating targeted geriatric screening programs (40). Period effects surged in India (2019 rate ratio [RR] = 1.213), Indonesia (RR = 1.22), and Saudi Arabia (RR = 1.228), likely driven by urbanization-linked pollution (e.g., unregulated wastewater in India’s Ganges Basin), dietary shifts toward processed foods (oil-driven economic expansion in Saudi Arabia), and fragmented primary care systems, where rural ultrasound coverage remains below 30%, delaying early diagnosis (10, 51, 52). In contrast, declining period trends in Russia (RR = 0.9056), Egypt, and globally reflect the success of tobacco control initiatives (Russia’s 2000s tax reforms reduced smoking prevalence by 15%) and the integration of screening measures (Egypt’s 2014 hepatitis C elimination program improved renal monitoring), though Russia’s high baseline mortality in older cohorts indicate the long-term repercussions of post-Soviet healthcare collapse (27). Cohort effects suggested generational divergence: Saudi post-1952 cohorts (RR > 1.37) faced risks from sedentary lifestyles and processed food dependence, while China’s post-1967 cohorts (RR = 1.1653) may bear the effects of industrial pollution and obesity epidemics, emphasizing the need for intergenerational nutrition interventions (3, 52). Hypothetically, nations demonstrating rising period effects may be experiencing systemic healthcare resource misallocation. India’s Ayushman Bharat scheme struggles with rural diagnostic gaps (<20% pathology access), whereas the UAE’s volatile data reflects the exclusion of migrant workers (80% population) from universal coverage, requiring employer-mandated insurance. Conversely, declining-period nations exemplify policy synergy: Russia’s tobacco taxation and non-communicable diseases programs reduced kidney cancer mortality by 0.7% annually, while China’s rural ultrasound projects have boosted early detection by 40%, yet urban PM2.5 exposure remains a latent threat (21, 39). Policy priorities should be context-specific. Brazil and India may benefit from mobile screening units (modeled on China) and food additive regulations (e.g., Indonesia’s proposed sugar tax), while Russia and Egypt could share tobacco control frameworks via BRICS health alliances. The global decline validates the list of carcinogens established by the WHO and cross-border screening. Cohort trends highlight the need for youth-centric interventions. Saudi Arabia could legislate school nutrition standards, while China must monitor the transgenerational impacts of industrial pollution exposure. Ultimately, APC modeling transcends risk stratification, advocating the importance of structural reforms. Ensuring equitable healthcare access, transnational data-sharing platforms, and enacting intergenerational health policies are pivotal to mitigating the kidney cancer burden.

Furthermore, the sharp rise in kidney cancer among people aged 80 and older in BRICS countries necessitates urgent reforms in health systems to provide geriatric-focused care, including the adoption of Geriatric Assessment to guide treatment decisions based on overall health rather than age alone, the involvement of geriatricians in multidisciplinary oncology teams to balance treatment efficacy with quality of life, the early integration of palliative care for symptom management and patient and family support, and the inclusion of geriatric cancer care within universal health coverage to ensure accessible and appropriate care—without which health systems may be overwhelmed by the complex needs of a growing older cancer population.

This study is subject to several limitations. Firstly, the accuracy and comparability of incidence and mortality estimates may be compromised by data incompleteness and variations in cancer registration practices across BRICS countries. Underdiagnosis in resource-limited regions, such as rural areas in India and Ethiopia, and potential overdiagnosis in affluent urban regions, such as those in the UAE and Saudi Arabia, could affect the observed trends. Secondly, while the intrinsic estimator method addresses collinearity within the APC model, it does not account for unmeasured confounders, including genetic susceptibility, environmental exposures, and socioeconomic factors, which may introduce bias into effect estimates. Furthermore, the APC model functions as a macro-level analytical tool and is not capable of directly quantifying the effects of specific risk factors, thus our interpretations remain inferential. Thirdly, national-level estimates obscure within-country disparities, such as urban–rural or socioeconomic differences, particularly in large and diverse nations like India and South Africa. Lastly, although the GBD estimates are designed for cross-national comparability, they may diverge from registry-based reports and are sensitive to the quality and coverage limitations of the underlying data.

## 5 Conclusion

The BRICS member countries exemplify the complex interplay between modernization and the rising burden of kidney cancer. While Russia’s healthcare reforms have demonstrated relative success in reducing mortality rates and serve as a model for integrated healthcare delivery, other BRICS member countries require urgent multisectoral strategies to address disparities in incidence and mortality. Prioritizing metabolic health through lifestyle interventions, implementing stringent environmental regulations to limit industrial pollution, and ensuring equitable access to early diagnosis and treatment are imperative amid ongoing demographic and epidemiological transitions. Context-specific policies, cross-national collaboration, and investments in healthcare infrastructure will be pivotal to mitigating the kidney cancer burden across these diverse populations.

## Data Availability

The original contributions presented in the study are included in the article/supplementary material, further inquiries can be directed to the corresponding author.

## Footnotes

^1^ https://vizhub.healthdata.org/gbd-results/
